# Evaluating the Views of Final-Year Medical Students Towards Their Medical Education Model: A Cross-Sectional Study

**DOI:** 10.7759/cureus.102942

**Published:** 2026-02-04

**Authors:** Mohammed Ahmed, Anisha Wakefield, Ahsan Siddiqui, Emily Dods, Deena D Vadiveloo, Ryan Mendes

**Affiliations:** 1 Medicine, University of East Anglia, Norwich, GBR; 2 Gastroenterology and Hepatology, Northern General Hospital, Sheffield, GBR; 3 General Surgery, East Surrey Hospital, Redhill, GBR; 4 General Medicine, Worcestershire Royal Hospital, Worcester, GBR

**Keywords:** final-year medical students, integrated model, medical learning, medical school education, spiral model, student education, traditional model

## Abstract

Introduction

Undergraduate medical education in the UK tends to follow one of three models: traditional, spiral, or integrated. This paper evaluates medical students' opinions of each model in terms of academic teaching, clinical teaching, and average satisfaction to determine which model is more effective at providing a well-rounded medical education.

Method

Final-year medical students were asked to fill in a survey evaluating the three models of medical education through three metrics: academic teaching, clinical teaching, and average satisfaction. Five universities were approached to recruit students: two employing the traditional model, two employing the spiral model, and one employing the integrated model. Responses were sought over a four-month period through email communication with final-year medical students. Responses were recorded using a 10-point ordinal scale.

Results

A total of 416 students' responses were included. The average score for academic teaching was 5.6 ± 1.4; the average score for clinical teaching was 5.4 ± 2.3; and the average satisfaction score was 5.6 ± 1.7. None of the models was significantly better or worse in academic teaching. The spiral curriculum was significantly better in clinical teaching (6.3 ± 1.1, p < 0.05), and the traditional model was significantly worse in clinical teaching (4.9 ± 2.2, p < 0.05).

Discussion

This novel paper demonstrates the views of final-year medical students towards the three models of medical education. None of the three models of education is superior in academic teaching, raising broader discussion points regarding how medical students absorb and learn medical content. We identify that early clinical exposure produces better clinical learning outcomes, with the integrated model acting as an effective baseline, from which institutes can look to build.

## Introduction

The evolution of medical education is difficult to truly formalise. The origins of medical education can be traced as far back as the Ancient Greeks, with an impromptu training regimen of what can archaically be called ‘see and do’ [1]. The Ancient Egyptians and Romans structuralised this informal learning pattern into what could be considered, in today's terms, an apprentice-like route to becoming a physician [2]. Indeed, the first remnants of medical training institutions can be traced back to Alexandria, Egypt [3], and Salerno, Italy [4], the origins of which pioneered European physician programmes, with the first formally established European medical school in Montpellier, France [5]. Since then, formal institutes responsible for training physicians have traversed the European continent, with the first formal medical institute in the United Kingdom being the University of Edinburgh in 1726 [6].

Since the inception of medical education in the United Kingdom, it has undergone several transitions; originally designed to be an apprentice-like model, it has evolved into a more formalised, degree-like approach since the 20th century. Though the exact origin of UK medical education is blurry, Clarke identifies five periods of transition, though it has likely since entered its sixth transition period [7]. The current route to attaining the title of ‘physician’ is to enter a university training programme and pursue five years of medical education, after which one can enter a residency programme. There has been some modernisation of this approach, with routes for six-year and four-year programmes, though the conventional approach remains a five-year university degree [8].

Within that five-year degree, there have also been significant changes to the structure that has been employed by these institutes. Though there is no formal date for the development of the first model of medical education, the earliest recognised model is the traditional model, conceptualised following the Flexner report of 1910 [9] and the Haldane report of 1913 [10]. This model was designed to have two separate phases: a preclinical phase and a clinical phase. Often, the university would dedicate three years of the degree to learning biomedical knowledge, anatomy, physiology, etc., without any clinical exposure, and then, in the final two years, there would be intense clinical exposure with minimal lecture-based learning [11]. This is the most commonly used curriculum presently. The second recognised model is the spiral curriculum, pioneered by the University of Dundee in 1995 [12]. This model was designed to integrate clinical exposure within the academic setting, with the student exposed to the practical application of theoretical knowledge immediately after it has been learned. This model is designed to help students build on the knowledge they learn in a ‘spiral’ fashion, simultaneously gaining depth of understanding in a topic and developing clinical skills. The most recent modernisation of medical education comes in the form of the integrated model, said to have been established in 2019 [13]. This model also integrates academic and clinical exposure; however, there is a heavier emphasis on academic learning in earlier years, with this ratio moving more positively towards clinical teaching in the latter years.

There has been little formalised work looking at how medical students feel towards the education they are receiving in the UK. Ben-Mabrouk et al. [14] have previously looked at how medical students felt towards their educators within the different models of education, and this study aims to build on that work. To our knowledge, there are no formal studies looking at how UK-based medical students feel about the models of medical education, though there is some work in other countries. Buja [15] looks at how the traditional model is perceived in the United States and discusses the burden of excessive academic learning in the preclinical years, without the ability to compound the learning with clinical exposure. Densen [16] looks at the challenges in implementing the spiral model of education, discussing the issues regarding the coordination and implementation of the model, with particular burdens on the hospital setting to accommodate large numbers of students, and the potential for students to lose out on valuable knowledge in pathophysiology, anatomy, and psychosocial areas of learning. This work looks to fill a gap in the literature, exploring the views of UK medical students towards the three models of medical education.

## Materials and methods

Population

The research protocol recruited final-year medical students enrolled in a professional medical degree programme from five medical schools. The five selected schools included two which follow traditional models, two which follow spiral models, and one which follows the integrated model. Medical schools were approached through email, and permission was obtained to distribute the questionnaire to their students. Upon agreement from the university, an email was circulated to all students containing the aims and objectives of the study and a link for participation. There was no target minimum number of students; however, based on previous experience, it was clear that the response rate would be low, and so an effort was made to ensure as many students were approached as possible. Of the five chosen universities, the estimated population of final-year medical students approximates 900. Responses were collected over a four-month period, with distribution starting in early October and collection ending towards the end of January, coinciding with the starting period of finals exams. There were no restrictions based on previous academic attainment or current year of study. The study was designed as a cross-sectional questionnaire. A sample size calculation of 416 participants was deemed necessary at a 0.95 power level on G*Power (version 3.1.9.7; Universität Düsseldorf, Düsseldorf, Germany).

Inclusion and exclusion criteria 

We assessed only current medical students enrolled in professional medical degrees and at one of the five chosen centres who provided their informed consent for the use of their data.

We excluded participants who had already completed their course, participants who had withdrawn from their medical degrees, participants not enrolled at one of the five selected centres, and participants who did not consent to the use of their data.

Questionnaire design

A cross-sectional, Association for Medical Educators in Europe (AMEE)-compliant questionnaire was distributed to participants (see Appendix A). In previous work from Ben-Mabrouk et al. [14] looking at undergraduate medical education, three metrics were utilised for assessing education: effectiveness of academic teaching, effectiveness of clinical teaching, and student satisfaction levels. We have chosen to use the same three metrics for assessment of this cohort, though some changes were made to the way these metrics were assessed. In their paper, the interpretation of the metrics was left broad; however, we have decided to narrow these parameters. This was achieved by asking students to fill in focussed subsection questions relating to the broader themes of effectiveness and satisfaction. These subsection scores were then averaged to give an overall score for each metric.

Questions were designed to utilise a 10-point ordinal scale, with the finite ends representing the extremes of satisfaction or dissatisfaction. The scale ranged from 0 to 10, with 0 representing complete ineffectiveness/complete dissatisfaction and 10 representing complete effectiveness/complete satisfaction. The three broad metrics were assessed in smaller subsections, the averages of which were totalled to give an overall average for each metric. The questionnaire underwent two-phase pretesting, with Phase 1 involving assessment and feedback from non-healthcare volunteers, and Phase 2 involving assessment and feedback from healthcare students from centres outside of the chosen ones for study. Prior to the two-stage pretesting system, an internal test was performed to assess for inconsistency. The rationale of pretesting in this fashion allowed for correction of issues regarding duration of application, language choice, and ambiguity in Phase 1, and allowed for feedback regarding applicability to the population and whether questionnaire design introduced bias in Phase 2.

Statistical analysis and outcomes 

The primary outcomes of this study were to determine the effectiveness of academic teaching, clinical teaching, and the satisfaction of medical students in each of the described models of undergraduate medical education. A secondary outcome was to assess the differences in medical students' views based on their enrolled model of education.

Data were entered into IBM SPSS Statistics for Windows, Version 30 (Released 2024; IBM Corp., Armonk, NY, USA), and analysis of the data was done using frequencies, proportions, group means, and standard deviations. Descriptive analysis was used to assess the different variables in the study.

The primary outcome was assessed through analysis of the entire data set, analysed by two assessors. This involved producing a mean value with a standard deviation for the entire population. Secondary outcomes were assessed via a range of measures, including analysis of the whole population to look for variation between year groups and the seniority of doctors. When assessing whether candidates' responses were eligible for inclusion in speciality data, responses were disregarded if there was a failure to answer all three metrics. Comparison of significance was done using paired t-testing, with a p-value of 0.05 or less deemed significant.

## Results

A total of 449 students responded to the survey, with 416 meeting the inclusion criteria. Students were grouped according to medical student teaching models: traditional (n = 188), spiral (n = 153), and integrated (n = 75), as shown in Table 1. The demographic summary table shows that 91% of students included were within the 24-26 age range, and 57% were school leavers.

**Table 1:** Demographic summary

Characteristic	Overall n = 416	Traditional n = 188	Spiral n = 153	Integrated n = 75
Age				
24-26	377 (90.63%)	181 (96.28%)	135 (88.24%)	61 (81.33%)
26-30	33 (7.93%)	4 (2.13%)	18 (11.76%)	11 (14.67%)
30+	6 (1.44%)	3 (1.60%)	0	3 (4.05%)
Previous educational attainment				
School leaver	237 (56.97%)	164 (69.20%)	73 (30.80%)	0
Intercalating student	136 (32.69%)	6 (4.41%)	58 (42.65%)	72 (52.94%)
Bachelor’s degree	32 (7.69%)	13 (40.63%)	19 (59.38%)	0
Master’s or higher	11 (2.64%)	5 (45.45%)	3 (27.27%)	3 (27.27%)

**Figure 1:**
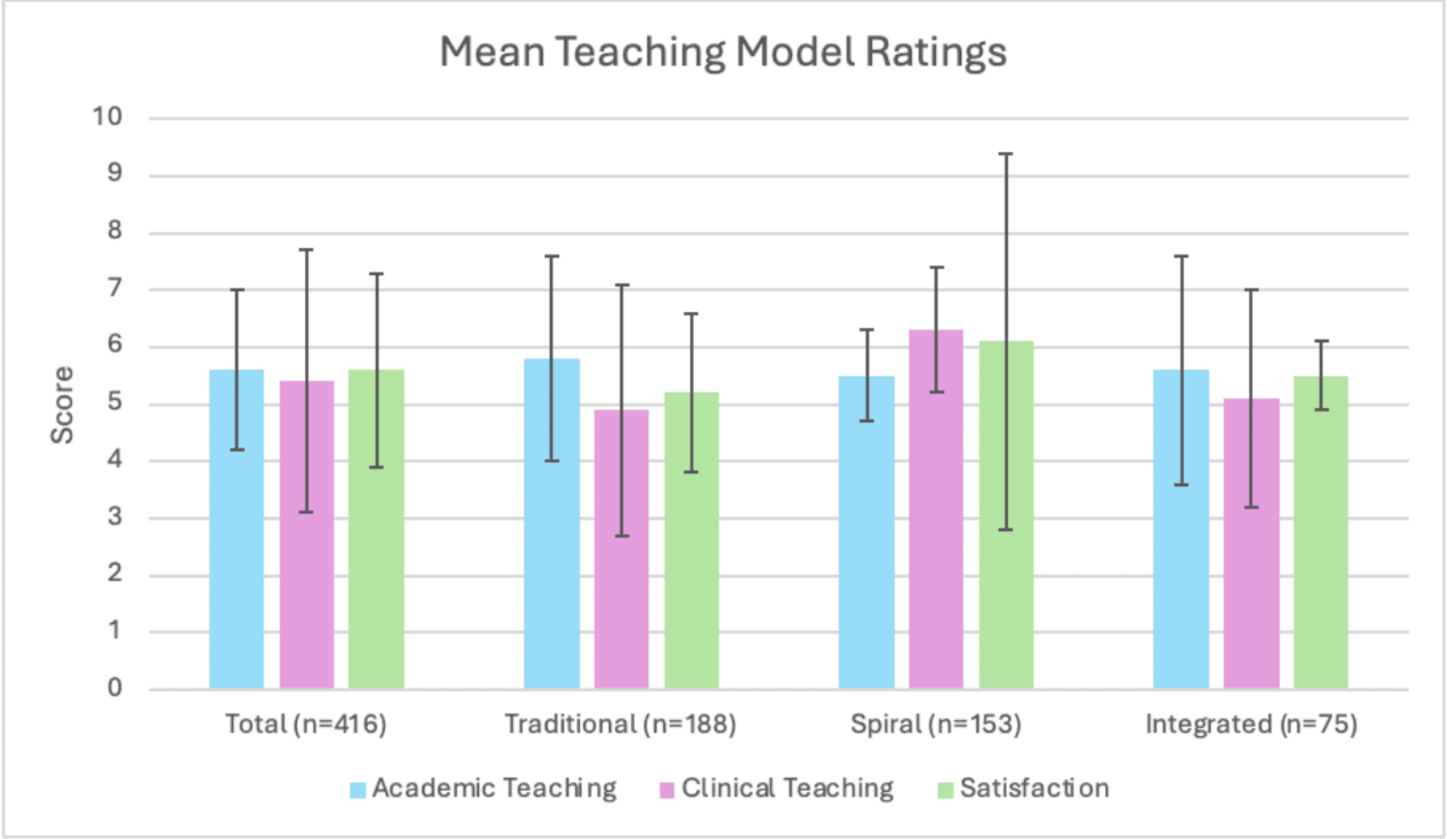
Comparison of means and standard deviations of the models of medical education across the three assessed metrics

Overall, academic and clinical teaching were rated similarly, with mean scores of 5.6 and 5.4, respectively, as shown in Table 2. Academic teaching had low variation between teaching models, whereas clinical teaching had more variation, with the spiral model given a mean rating of 6.3 compared to 4.9 and 5.1 in the traditional and integrated groups. Comparison between the models of education and SD can be seen in Figure 1, and the significance between models can be seen in Table 3. 

**Table 2:** Summary of mean ratings from a 10-point ordinal scale, organised by teaching model * represents significance at p < 0.05.

Type of educational model	Number of responses	Average academic teaching score	p-value	Average clinical teaching score t-test	p-value	Average satisfaction score	p-value
Traditional	188	5.8 ± 1.8	0.1388	4.9 ± 2.2*	0.0124	5.2 ± 1.4*	0.0049
Spiral	153	5.5 ± 0.8	0.4043	6.3 ± 1.1*	0.0001	6.1 ± 3.3*	0.0188
Integrated	75	5.6 ± 2.0	1.000	5.1 ± 1.9	0.2871	5.5 ± 0.6	0.6149

**Table 3:** Comparison of p-values between metrics of medical education models * represents significance at p < 0.05.

Intramodel comparisons	Academic teaching	Clinical teaching	Satisfaction
Traditional vs Spiral	0.0566	0.0001*	0.0001*
Traditional vs Integrated	0.4135	0.4902	00747
Spiral vs Integrated	0.5913	0.0001*	0.1201

Within academic teaching, applicability of teaching to real-world settings was rated the highest (5.8 ± 0.7), while preparedness for finals exams was rated less favourably (4.8 ± 1.9). Confidence in answering questions in a hospital setting scored 5.5 ± 2.4. In clinical teaching, confidence in managing clinical scenarios scored highly (5.9 ± 1.0), while preparedness for OSCE/practical exams (5.0 ± 1.1) and foundation training (5.4 ± 2.1) were rated slightly lower.

Satisfaction with teaching from lecturers and clinical placements was consistent throughout the population, with mean scores of 5.5 ± 1.3 and 5.5 ± 1.8, respectively. The statement ‘I am satisfactorily prepared to be a doctor’ had a mean score of 5.6 ± 0.4, showing little variation in responses.

Spiral teaching students showed the highest overall satisfaction (mean 6.1); however, this group also had the greatest variability in satisfaction scores, with a standard deviation of 3.3, suggesting a broader range of experiences within the population. 

## Discussion

To the best of our knowledge, this paper is the first of its kind looking at how UK medical students view the different models of medical education, and the first of its kind in comparing the three models. We build on work by Ben-Mabrouk et al. [14], using the same three metrics to assess final-year medical students’ perceptions of the model of education employed by their institute. We show that students showed no significant preference towards the academic teaching they were receiving in any model of education, and that clinical teaching and satisfaction were significantly better in the spiral model. The data also show that there is broad variation in the views of the students, best seen in the wide standard deviations in all of the metrics across all of the groups.

Analysis of the results of this study raises some interesting discussion points. Given that there is no significant difference in academic teaching scores between the models, and all three models have approximately the same overall score, this raises an interesting discussion as to how students are being educated. Previous work by Densen [16] has discussed the difficulty in actually teaching medical knowledge to students. There have been numerous studies looking at how the traditional lecture environment is an inefficient model of learning, with this method demonstrating the lowest retention of knowledge, but there seems to be no better alternative to the lecture environment [17,18]. There is an understated understanding within medical institutions that medical students will naturally find extra resources to learn, and that often it is these extracurricular materials that most students rely on to pass exams and gain academic knowledge. These factors perhaps best explain why academic learning scores are very similar in all three models, as it is not the model itself that influences academic learning, but rather the educator and the limitations of the delivery of knowledge. The elimination of the traditional lecture has to be considered carefully, especially with the expansion of medical student numbers, as other methods may struggle to deliver the scope of knowledge needed for medicine to such a large cohort.

The clinical metric raises a wider and more pertinent discussion point. Our work shows that the spiral model is significantly better in clinical education, and that the traditional model is significantly worse. This view towards clinical teaching is not uncommon in academia, and a review by Shariati et al. [19] shares similar findings. It is perhaps naive to suggest that this outcome is solely due to the earlier exposure to the clinical setting, because while this does contribute, given that the integrated model is not also significantly better, there must be additional elements to this learning. The significant improvement in clinical learning in the spiral model is likely a multifactorial response to receiving academic learning and then immediately receiving the supplementary clinical learning. It is almost intuitive, and indeed proven in academia, to suggest that having the opportunity to immediately apply academic knowledge in real clinical settings helps build memory aids and provides students with real-world reference points to which theoretical knowledge applies [20]. This limitation of the traditional model, with the denial of clinical exposure in the early years, has been extensively discussed in the literature. Hashemiparast et al. [21] explore this in their work and reach similar conclusions, examining the restriction of applying theoretical knowledge early. The issue with these models is that, where they lack in one domain, they contribute in others, and it is often suggested that the spiral model is difficult to integrate, as there are often gaps in students’ knowledge that can be deemed less useful clinically [15].

The integrated model of education is difficult to discuss in detail, as, at the time of writing, there is only one university that identifies itself as a truly integrated model. In theory, this model is perhaps the best placed to educate students, providing a hybrid of academic and clinical exposure. Indeed, there is a growing shift in traditional universities applying a makeshift integrated system, with some early exposure in general practice or outpatient clinics in the early years to help students get a taste of clinical exposure. From our data, it can almost be suggested that the integrated model is the baseline; all three metrics of the integrated model are non-significant from the global average. This is interesting, as it raises an ambiguous discussion around what constitutes the best teaching a medical student can achieve. Medical education in the UK has an extremely low attrition rate, with an estimated 5% of all final-year students failing their final exams, and, of that small population, an estimated 92% had extenuating circumstances that contributed to their failing. Therefore, there is an argument that it does not matter which model is employed by a university, as the number of medical students graduating continues to remain satisfactory. There are considerable other challenges to medical education, with between 20% and 35% of students considering dropping out of medical education [22]. Indeed, there are great challenges regarding the responsibility placed on these institutes to educate aspiring doctors. Perhaps by recognising baselines such as the integrated model, universities can be provided with a framework from which they can build. There is no need to have one absolute model of learning; however, it is becoming increasingly apparent that a hybrid of academic and clinical learning is the most effective strategy for learning medicine.

We recognise that this work, like all works, has its limitations. We made an intentional decision to assess only final-year medical students who enrolled in a five-year training programme, as we felt this would be the best reflection of the data we wished to gather; however, we recognise that this decision does exclude some vital voices in the discussion, such as postgraduate learners. We also recognise that by only selecting final-year medical students, we exclude the changing perceptions of students throughout their learning in their own model. This is perhaps a work that may be of value in future academia - looking at a selected group of students in the models of medical education and exploring their changing perceptions throughout the years of their learning. Another limitation of this paper is the design: a qualitative study. There has been extensive work examining the limitations of qualitative work and low turnout in such papers; however, this method remains the most effective in collecting such data [23]. There remains no better way to assess the views of such groups outside of qualitative work. The metrics we used are similar to those used by Ben-Mabrouk et al. [14]; however, we expand on them by introducing micro-scoring systems within the broader domains, though, like them, we note that these metrics are difficult to objectify.

## Conclusions

Overall, this work is a novel discussion regarding the views of medical students towards the three models of medical education. We have explored that academic learning is largely similar between any model of medical education, and that clinical learning is always better when there is early clinical exposure. This work looks to fill a vital gap in the perceptions of medical students towards the education they receive and exposes further gaps that we hope other academics will endeavour to fulfil. 

## Appendices

Appendix A

Section 1: Consent

We appreciate and thank you for taking the time to complete this form. The questionnaire will act as the foundation for our research, looking into how effective NHS consultants have been teaching medical students.

We would like to inform you that by continuing, you are consenting to having your data stored for an indefinite period of time, to allow for analysis and further research.

Your data will be stored in accordance with GDPR guidelines and governmental data regulations.

If at any later point in time you wish for your consent to be revoked, please email us at: nhsconsultantresearch@gmail.com

- I hereby consent to the use of my data. (continue to next section) - I do not consent to the use of my data. (end form)

Section 2: Stratification Questions

I am a current final year medical student - Yes - No (end form) - I am currently intercalating/ taking a break/ have withdrawn (end form)

What was your highest level of qualification at the time of admission to medical school? - School leaver - Bachelor’s degree - Master’s degree - PhD/ other professional degree

Which of the below Medical Schools are you currently enrolled in? - Norwich Medical School - Imperial School of Medicine - Sheffield Medical School - Hull-York Medical School - Southampton Medical School - My Medical School is not listed here

What is the current model of teaching employed by your medical school? - Problem-Based Learning (PBL) - Traditional - Integrated

Section 3: Average Effectiveness of Academic Teaching

The following questions will ask you to rate your formal timetabled teaching on a scale of 0-10; where 0 is absolutely disagree and 10 is completely agree.

When considering your answers, please only take into account formal timetabled lectures and seminars.

1. I feel like my timetabled teaching throughout medical school has effectively prepared me for passing my medical exams a. 10-point ordinal scale

2. I feel like my timetabled teaching sessions throughout medical school have adequately developed my knowledge of medical conditions a. 10-point ordinal scale

3. I think my formal timetabled teaching sessions have been effective to my learning a. 10-point ordinal scale

4. I feel like my academic teachers are good at teaching me about medicine a. 10-point ordinal scale

5. I think the teaching material is effective in preparing me to become a doctor a. 10-point ordinal scale

6. I think my university curriculum is effective a. 10-point ordinal scale

Section 4: Average Effectiveness of Clinical Teaching

The following questions will ask you to rate your formal allocated clinical exposure throughout medical school on a scale of 0-10, where 0 is absolutely disagree and 10 is completely agree.

When considering your answers, please only take into account formal timetabled clinical teaching and placement.

1. I feel like I have had adequate exposure to the hospital setting throughout my medical education a. 10-point ordinal scale

2. I feel like I have had enough exposure to all the specialties within the hospital a. 10-point ordinal scale

3. I feel like my clinical exposure has helped me to apply my theoretical knowledge to real patients a. 10-point ordinal scale

4. I think my clinical teaching has been effective in preparing me to become a doctor a. 10-point ordinal scale

5. I am happy with the amount of clinical exposure I received in medical school a. 10-point ordinal scale

6. I learned more from being in the hospital than outside of the hospital a. 10-point ordinal scale

Section 5: Average Satisfaction Throughout Medical School

1. I am satisfied with the academic teaching I have received throughout medical school a. 10-point ordinal scale

2. I am satisfied with the clinical teaching I have received in medical school a. 10-point ordinal scale

3. I am satisfied with my medical school a. 10-point ordinal scale

Section 6 (Optional): NHS Consultants as Teachers Overall

The next set of questions is all optional, but we would like to understand what specifically made you choose your ratings. Please provide a few words or a sentence describing your views on the following matters:

1. Think of the most effective consultant-led teaching you’ve received. What specifically makes it memorable? a. Qualitative response

2. Think of some consultant-led teaching which was not as effective as you hoped. What about it was not effective, and how would you like it improved? a. Qualitative response

3. If you do not attend formal teaching or you find formal teaching less effective, how do you prepare for your end-of-year exams? (this may be: YouTube videos, independent textbook reading, OSMOSIS, independent group work, etc.) a. Qualitative response

Section 7: Comparing Seniority With Effectiveness in Teaching

The next set of questions is a series of yes/no questions.

1. I think that NHS consultants should be teaching medical students a. Yes/no

2. I find that the teaching from less senior doctors is more effective than that from consultants a. Yes/no

3. I find the teaching from other healthcare professionals (nurses, dietitians, midwives, etc) is more effective than that from consultants a. Yes/no

Section 8 (For Forced End of Form)

If you have ended up here, it means you were not part of the population we had intended to research. There was no error in your answers, so please don't resubmit using different answers. We thank you for your time, nonetheless.

If you would like further information on who we were testing and why your answers may have resulted in you being excluded, please feel free to email: nhsconsultantresearch@gmail.com.

Section 9 (For Successful Completion of Form)

Thank you for completing our questionnaire. Through you and hundreds of other medical students, we hope to learn more about how your education has been developing. We know that medical school is incredibly difficult and in no way perfect, and through this research, we hope to evaluate one of the key stresses in medical school: your education. We wish all of you luck in your future education, no matter what stage you are currently at; you are the future of healthcare.

If you would like any further information about this research paper, including how your data will be stored, processed and used to create results, please feel free to email nhsconsultantresearch@gmail.com.
